# Targeting RTKs/nRTKs as promising therapeutic strategies for the treatment of triple-negative breast cancer: evidence from clinical trials

**DOI:** 10.1186/s40779-024-00582-z

**Published:** 2024-12-12

**Authors:** Kasshish Mehta, Mangala Hegde, Sosmitha Girisa, Ravichandran Vishwa, Mohammed S. Alqahtani, Mohamed Abbas, Mehdi Shakibaei, Gautam Sethi, Ajaikumar B. Kunnumakkara

**Affiliations:** 1 Cancer Biology Laboratory, Department of Biosciences and Bioengineering, Indian Institute of Technology Guwahati (IITG), Guwahati, Assam 781039 India; 2 Radiological Sciences Department, College of Applied Medical Sciences, King Khalid University, 61421 Abha, Saudi Arabia; 3 BioImaging Unit, Space Research Centre, Michael Atiyah Building, University of Leicester, Leicester, LE1 7RH UK; 4 Electrical Engineering Department, College of Engineering, King Khalid University, 61421 Abha, Saudi Arabia; 5 Department of Human-Anatomy, Musculoskeletal Research Group and Tumor Biology, Chair of Vegetative Anatomy, Institute of Anatomy, Ludwig-Maximilian-University, 80336 Munich, Germany; 6 Department of Pharmacology, Yong Loo Lin School of Medicine, National University of Singapore, Singapore, 117600 Singapore; 7 NUS Centre for Cancer Research (N2CR), Yong Loo Lin School of Medicine, National University of Singapore, Singapore, 117699 Singapore

**Keywords:** Triple-negative breast cancer (TNBC), Tyrosine kinase, Clinical trial, Personalised medicine, Genetic diversity, Patient stratification

## Abstract

The extensive heterogeneity and the limited availability of effective targeted therapies contribute to the challenging prognosis and restricted survival observed in triple-negative breast cancer (TNBC). Recent research indicates the aberrant expression of diverse tyrosine kinases (TKs) within this cancer, contributing significantly to tumor cell proliferation, survival, invasion, and migration. The contemporary paradigm shift towards precision medicine has highlighted TKs and their receptors as promising targets for pharmacotherapy against a range of malignancies, given their pivotal roles in tumor initiation, progression, and advancement. Intensive investigations have focused on various monoclonal antibodies (mAbs) and small molecule inhibitors that specifically target proteins such as epidermal growth factor receptor (EGFR), vascular endothelial growth factor (VEGF), vascular endothelial growth factor receptor (VEGFR), cellular mesenchymal-epithelial transition factor (c-MET), human epidermal growth factor receptor 2 (HER2), among others, for combating TNBC. These agents have been studied both in monotherapy and in combination with other chemotherapeutic agents. Despite these advances, a substantial terrain of unexplored potential lies within the realm of TK targeted therapeutics, which hold promise in reshaping the therapeutic landscape. This review summarizes the various TK targeted therapeutics that have undergone scrutiny as potential therapeutic interventions for TNBC, dissecting the outcomes and revelations stemming from diverse clinical investigations. A key conclusion from the umbrella clinical trials evidences the necessity for in-depth molecular characterization of TNBCs for the maximum efficiency of TK targeted therapeutics, either as standalone treatments or a combination. Moreover, our observation highlights that the outcomes of TK targeted therapeutics in TNBC are substantially influenced by the diversity of the patient cohort, emphasizing the prioritization of individual patient genetic/molecular profiles for precise TNBC patient stratification for clinical studies.

## Background

Breast cancer has emerged as a prevailing malignancy, surpassing even lung cancer in terms of prevalence [1, 2]. This form of cancer constitutes 11.7% of all reported cancer cases, and its impact is profound, claiming 684,996 lives solely in the year 2020. As the foremost cause of cancer-related mortality among women globally, breast cancer is a critical health challenge [1–3]. Within this broader category, a subset of cases (15–20%) is known as triple-negative breast cancer (TNBC), marked by its aggressive and heterogeneous nature [4–8]. TNBC exhibits a poor prognosis, increased likelihood of recurrence, distant metastasis, and disease progression due to the absence of effective treatment options [4–8]. Remarkably, TNBC contributes to 90% of breast cancer-related mortalities, with elevated incidence rates observed among women of Indian (22–43%) or African (20–79%) descent, and an inclination toward young women [2, 3, 7, 9]. The conventional therapeutic landscape for TNBC entails a combination of chemotherapy, radiation therapy, and surgery [4]. Anthracyclines and taxanes often constitute first-line therapy for TNBC; however, these treatments are accompanied by undesirable side effects, such as cardiotoxicity [10]. Moreover, chemoresistance within a subset of patients poses additional challenges [7]. The current potential treatment strategies for this cancer encompass immunotherapy, chemotherapy, antibody–drug conjugates (ADCs), and targeted therapies [5, 10, 11]. These potential interventions, include tyrosine kinase inhibitors (TKIs), poly (ADP-ribose) polymerase (PARP) inhibitors, immune checkpoint inhibitors, and androgen receptor antagonists. While certain targeted therapeutics like olaparib, pembrolizumab and atezolizumab have received approval from the Food and Drug Administration (FDA) for the therapeutic management of TNBC, several interventions are currently being subjected to clinical scrutiny for their efficacy, safety, and economic viability [5, 10–14].

Significantly, TKs, constituting a subgroup of proteins orchestrating the activation of receptor TKs (RTKs), assume a central and indispensable role in the regulation of cellular proliferation and functionality [15, 16]. Perturbations in the equilibrium of these proteins have been strongly associated with unbridled cell proliferation, circumvention of programmed cell death, and the facilitation of angiogenesis within neoplastic growths [17]. Disturbed signaling pathways pivotal for cell survival and resistance to chemotherapeutic agents, notably the phosphoinositide 3-kinase (PI3K)/Akt and mammalian target of rapamycin (mTOR) pathways, frequently manifest irregularities within the subset of TNBC, closely aligned with the dysregulation of RTKs. Vigorous exploration is underway concerning targeted therapeutic interventions directed towards these oncogenic pathways, and promising therapeutic outcomes have emerged [6, 11, 18]. Remarkably, TK targeted therapeutics have manifested clinical efficacy by obstructing the aberrant pathways governed by these kinases [19]. This particular classification of therapeutics, encompassing both mAbs and small molecular agents, is presently the subject of comprehensive investigation within clinical trials. The ramifications extend beyond TNBC treatment, encompassing different cancer types [19–22]. This review aims to provide an overview of the various TKs which are potential drug targets in TNBC patients and summarises the clinically impactful results of the existing TK targeted therapeutics.

## TKs

TKs constitute a distinct subset of proteins that facilitate the phosphorylation of tyrosine residues within proteins, utilizing adenosine triphosphate molecules as a phosphate source [23, 24]. These enzymes exert a pivotal role in the operation of signal transduction pathways, thereby influencing essential cellular processes encompassing cell survival, proliferation, and related metabolic activities [25–27]. The classification of TKs is dichotomized into two categories based on their functional attributes and cellular localizations: RTKs and non-RTKs (nRTKs) [25]. nRTKs are intracellular TKs contingent upon RTKs for activation, thereby orchestrating diverse biological events associated with the acquisition of cancer hallmarks [19, 28]. In contrast, RTKs represent transmembrane cell surface receptors characterized by a tripartite domain structure encompassing an extracellular moiety, a transmembrane segment, and a cytosolic region harboring the TK catalytic domain [29, 30].

A panoply of RTKs and nRTKs, including but not limited to platelet-derived growth factor receptor (PDGFR), epidermal growth factor receptor (EGFR), fibroblast growth factor receptor (FGFR), vascular endothelial growth factor receptor (VEGFR) family, anexelekto (AXL), insulin-like growth factor receptor (IGFR), mesenchymal-epithelial transition factor (MET), etc., play pivotal roles in fostering hyperproliferation, angiogenesis, evasion of apoptosis, cellular invasion, tumor cell motility, tumor advancement, metastasis, and resistance to therapeutic agents [24]. These RTKs and nRTKs interlace with vital signaling pathways, such as PI3K/Akt, signal transducer and activator of transcription 3 (STAT3), rat sarcoma (Ras)-mitogen-activated protein kinase (MAPK), and phospholipase C-gamma (PLC-γ)/protein kinase C (PKC), and any aberrations therein, including DNA amplification, activating mutations within RTK proteins, dysregulated RTK ligands, or fusion events involving RTKs, have been implicated in the initiation and progression of oncogenic processes [19, 29–31]. For example, instances of mutated *EGFR* accompanied by ligand amplification have been discerned in breast cancer, colorectal cancer, cutaneous squamous cell carcinoma, and non-small cell lung cancer (NSCLC) [30–34]. Moreover, overexpression of cellular MET (c-MET)/MET has been documented in diverse malignancies, including NSCLC, hepatocellular carcinoma, gastroesophageal cancer, and gliomas, further emphasizing their role in oncogenesis [29, 35–37]. Likewise, perturbations in the regulation of RTK ligands have been observed in various carcinomas such as medullary thyroid, breast, bladder, and gastrointestinal stromal tumors [29, 31].

It is noteworthy that more than 30% of cancer cases exhibit dysregulated or mutated RTKs, rendering these proteins significant targets for precision therapies [24]. Substantiating this, a spectrum of mAbs and small molecule inhibitors have gained approval from the FDA for therapeutic interventions in diverse malignancies. Small molecule TKIs, exemplified by afatinib, alectinib, crizotinib, etc., have found utility in managing NSCLC, while sorafenib, pazopanib, sunitinib, among others, have been sanctioned for treating renal cell carcinoma, and bosutinib, imatinib, ponatinib, etc., have demonstrated efficacy in treating chronic myeloid leukemia, primarily targeting distinct RTKs [38–41]. In parallel, numerous mAbs, including trastuzumab and pertuzumab targeting human epidermal growth factor receptor 2 (HER2), cetuximab and panitumumab targeting EGFR, and bevacizumab targeting vascular endothelial growth factor (VEGF), have been recommended for therapeutic use in conditions such as colorectal cancer, head and neck squamous cell carcinoma (HNSCC), breast cancer, and NSCLC [42–44]. As personalized and combinatorial therapeutic strategies burgeon in the realm of cancer management, strategies involving TK targeted therapeutics present a promising avenue, manifesting remarkable clinical outcomes across a spectrum of carcinomas.

## RTKs and nRTKs in TNBC

**Fig. 1:**
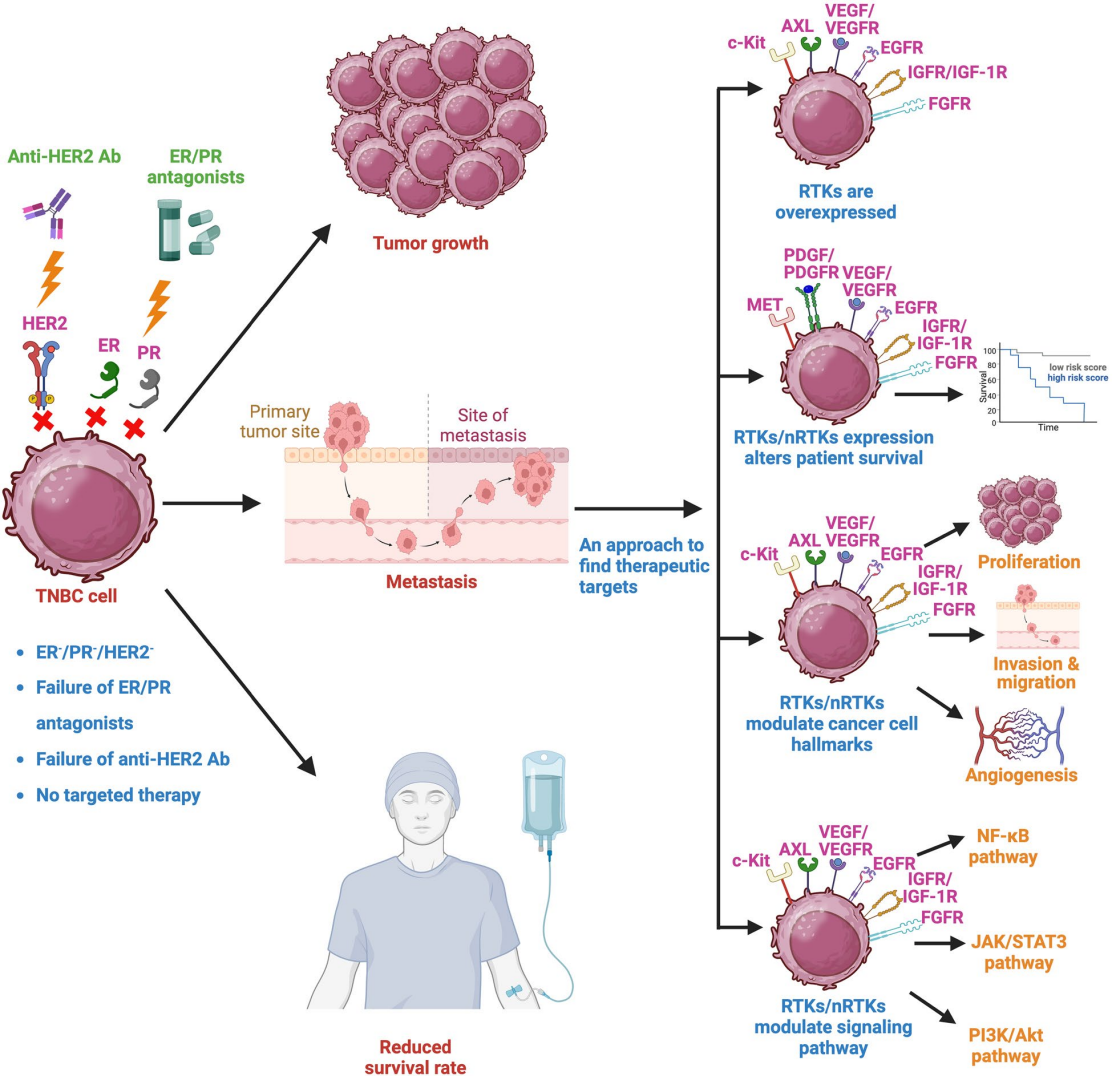
RTKs and nRTKs are deregulated in TNBC patients. TNBC, a highly aggressive breast cancer subtype, lacks expression of estrogen receptor (ER), progesterone receptor (PR), and human epidermal growth factor receptor 2 (HER2). Traditional therapies targeting these receptors are ineffective against TNBC, leading to tumor progression, metastasis, and decreased survival rates. Consequently, research efforts aimed at identifying alternative treatments have focused on understanding the dysregulated signaling pathways in TNBC cells. This approach has revealed the significant deregulation of TKs within TNBC. Among these, AXL, c-Kit, EGF/EGFR, FGF/FGFR, IGF/IGFR, MET, PDGF/PDGFR, and VEGF/VEGFR have been identified as overexpressed proteins associated with poor survival outcomes in TNBC patients. Importantly, these aberrant RTKs and nRTKs play crucial roles in regulating cellular processes, including signaling cascades, proliferation, angiogenesis, invasion, and migration, rendering them viable targets for therapeutic intervention. RTKs receptor tyrosine kinases, nRTKs non-receptor tyrosine kinases, TNBC triple-negative breast cancer, EGFR epidermal growth factor receptor, IGFR insulin-like growth factor receptor, IGF-1R insulin-like growth factor 1 receptor, TKs tyrosine kinases, MET mesenchymal-epithelial transition factor, PDGFR platelet derived growth factor receptor, VEGFR vascular endothelial growth factor receptor, Ab antibodies, AXL anexelekto, NF-κB nuclear factor kappa B, JAK/STAT janus kinase/signal transducers and activators of transcription, PI3K phosphoinositide 3-kinase

The genetic analysis of patient data with TNBC has revealed distinct subtypes, such as basal-like 1 (BL1), basal-like 2 (BL2), mesenchymal (M), mesenchymal stem-like (MSL), immunomodulatory (IM), and luminal androgen receptor (LAR) subtypes [45]. Research has also uncovered regional genetic variations within TNBC biopsies from different patients [46]. This genetic diversity contributes to the ineffectiveness of current TNBC treatments, as evidenced by the failure of targeted therapy using trastuzumab in patients with low HER2 expression [47]. Furthermore, the impact of this heterogeneity on treatment resistance has been confirmed through a clinical trial involving 579 breast cancer patients. This trial showed that the combination of lapatinib (an inhibitor of EGFR and HER2) with paclitaxel did not provide significant benefits to TNBC and HER2^−^progesterone receptor (PR) high patient groups but exhibited notable therapeutic advantages in other metastatic breast cancer cohorts [48, 49]. Interestingly, the same trial reported an antagonistic effect of lapatinib when combined with paclitaxel in the HER2^−^/PR^−^ subgroup [48, 49]. Characterized by such a remarkable and challenging degree of heterogeneity along with frequent recurrence, metastatic propensity, and an unfavorable disease prognosis, TNBC presents a daunting therapeutic challenge, as it lacks approved targeted interventions endorsed by the FDA, consequently relying heavily on chemotherapy for management [50–53]. Nevertheless, recent investigations have unveiled the pivotal involvement of RTKs and nRTKs in instigating, advancing, and propelling the progression of TNBC, thereby accentuating the potential utility of therapeutics targeting these molecules either alone or in conjunction with other therapeutic agents [50, 51, 54, 55]. RTKs and nRTKs that are deregulated in TNBC patients are illustrated in Fig. 1.

Primarily, a diverse array of RTKs and nRTKs, encompassing c-Kit, AXL, VEGF, EGFR, IGFR, FGFR, récepteur d’origine nantais (RON), MET and others, have been reported to be prominently overexpressed in numerous patients afflicted with TNBC, thereby playing a decisive role in shaping the disease prognosis [17, 51, 53, 56–61]. Secondly, the expression of certain RTKs/nRTKs, including VEGF, MET, EGFR, PDGF/PDGFR, IGF-1R, and FGFR has been correlated with diminished overall survival (OS). In addition, escalated VEGF expression aligns with curtailed progression-free survival (PFS), and elevated PDGF-C is suggestive of impaired distant-metastasis-free survival in TNBC patients [51, 57, 60, 62–68]. Thirdly, numerous RTKs/nRTKs, particularly those belonging to the realm of growth factor receptors, contribute to the sustenance of TNBC cell survival and proliferation, concurrently orchestrating the evasion of apoptosis, thereby significantly fostering the tumorigenicity of TNBC cells [51, 68–70]. Fourthly, a spectrum of RTKs/nRTKs, comprising PDGFR, VEGFR, FGFR-1, EGFR, AXL, Src, and c-Kit, have been implicated in inducing TNBC cell invasion and migration, and enhancing exit from the primary tumor microenvironment [2, 50, 53, 55, 60, 68, 71]. Fifthly, compelling associations between the overexpression of VEGF and its cognate receptor and the induction of angiogenesis and vascular permeability in TNBC have been discerned [66, 72, 73]. Further, a multiplicity of RTKs, prominently featuring EGFR, IGF-1R, and PDGF-C/PDGFR, alongside their intricate signaling pathways including nuclear factor kappa B (NF-κB), janus kinase/signal transducers and activators of transcription (JAK/STAT), and phosphatase and tensin homolog (PTEN)/PI3K/Akt/mTOR, have been implicated in fostering augmented chemo-resistance in patients afflicted by TNBC [51, 68]. Importantly, diverse signaling pathways and molecular interaction including RTKs/nRTKs like WEE1 and EGFR, play a role in conferring resistance to radiation therapy in TNBC [74]. In this context, the dysregulation in the expression of these proteins signifies an augmented risk of tumor recurrence and disease relapse [66, 68].

Numerous studies have elucidated that alterations particularly overexpression of RTKs/nRTKs contribute to therapeutic resistance in breast cancer patients. For example, overexpression of EGFR has been documented as a predictive factor influencing the response to trastuzumab in HER2^+^ breast cancer [75]. Additionally, deregulated expression of EGFR in the inducible form, *v-ERb-B:ER* gene was shown to confer both chemo- and radio-resistance in breast cancer cells [76]. In another study, overexpression of ceramide synthase 6 (CERS6) was found to be associated with chemo-resistance in TNBC patients and mechanistically, CERS6 was found to confer chemo-resistance by upregulating EGFR [77]. Also, overexpression of EGFR is observed in 64% of patients with TNBC. Copy number variation in EGFR has been strongly correlated with poor clinical outcomes in this patient population [78]. A recent investigation delineated that tumors characterized by elevated tyrosine protein kinase receptor 3 (TYRO3) expression manifest resistance to anti-programmed cell death protein 1 (PD-1)/PD-L1 interventions in both murine models and human subjects. Notably, the suppression of TYRO3 was found to induce tumor ferroptosis, thereby sensitizing resistant tumors to anti-PD-1 therapy [79].

The augmented expression of truncated forms of RTKs/nRTKs in neoplastic tissues has been implicated as a causative factor in therapeutic resistance. Specifically, p95HER2, alternatively referred to as p95HER2/611 carboxyterminal fragment or p110, constitutes a truncated variant of the HER2 receptor. This variant is generated either through the proteolytic shedding of the extracellular domain of HER2 or via translation initiation of *HER2* mRNA at internal codons [80, 81]. Empirical evidence from prior investigations signifies the prognostic value of p95HER2 expression, with enhanced levels correlating with unfavorable clinical outcomes. Additionally, elevated p95HER2 expression is associated with a more aggressive disease phenotype [80, 82, 83]. Notably, a clinically elevated p95HER2/HER2 ratio has been identified as being linked to poor outcomes in trastuzumab-treated HER2-positive metastatic breast cancer, highlighting the involvement of this truncated form in therapeutic resistance against trastuzumab [84–86]. Indeed, therapeutic interventions involving lapatinib have demonstrated efficacy in addressing individuals characterized by the overexpression of p95HER2, who do not experience benefits from trastuzumab treatment [87–89]. Hence, the identification of specific forms of RTKs/nRTKS expressed in patients is imperative for informed therapeutic decision-making. This approach holds the potential to optimize treatment strategies, resulting in time savings, improved personalized care, and enhanced survival outcomes. In addition, T cell bispecific p95HER2 antibodies have exhibited efficacy in diminishing the growth of p95HER2-expressing primary breast tumors in patient-derived xenograft models [85]. Nonetheless, the imperative for tailored clinical trials is necessary to effectively leverage anti-p95HER2 therapies.

Cumulatively, the intricate involvement of RTKs/nRTKs in activating signaling cascades and molecular mechanisms pivotal for the acquisition of diverse cancer hallmarks in TNBC patients has come to the fore. Consequently, the inhibition of these molecules emerges as a promising avenue in the therapeutic landscape against TNBC.

## TK targeted therapeutics in clinical trials for TNBC

**Fig. 2:**
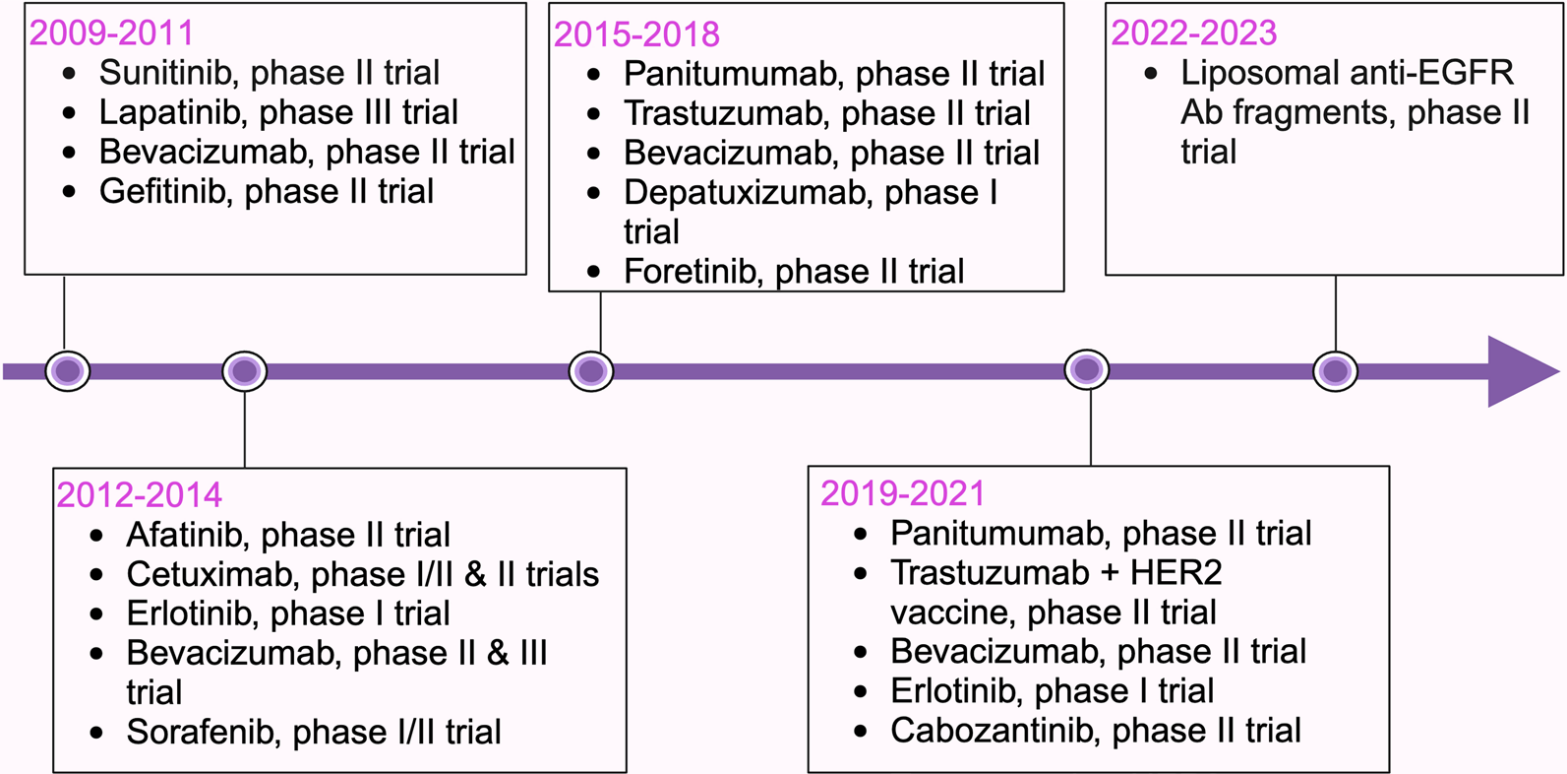
The historical account of RTK and nRTK targeted therapeutics utilized across different phases of clinical trials in patients with TNBC. In clinical trials targeting TNBC, inhibition of RTKs and nRTKs has been a focal point. The trials have explored diverse methods, employing monoclonal antibodies, small molecule inhibitors, and nanoparticles. The accompanying figure showcases a range of RTK and nRTK targeted therapeutics, such as sunitinib, lapatinib, gefitinib, afatinib, erlotinib, sorafenib, cabozantinib, foretinib, cetuximab, bevacizumab, panitumumab, depatuxizumab, and trastuzumab, utilized at various stages of these clinical trials. RTK receptor tyrosine kinase, nRTK non-receptor tyrosine kinase, TNBC triple-negative breast cancer, HER2 human epidermal growth factor receptor 2, EGFR epidermal growth factor receptor

**Fig. 3:**
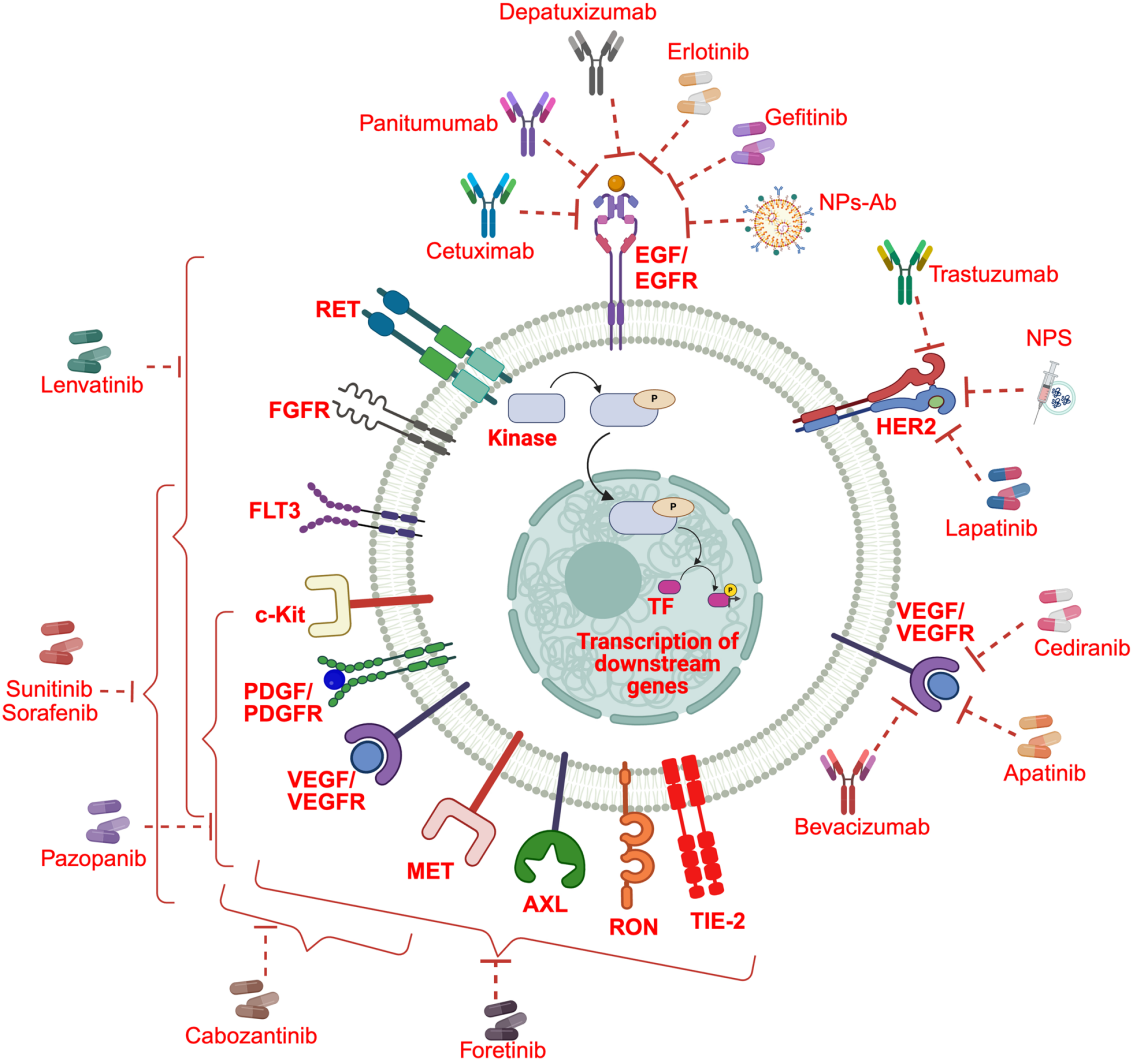
Clinical utilization of TKIs and RTKIs in TNBC. The RTK and nRTK targeted therapeutics employed in clinical trials for TNBC treatment act via specific molecular targets. Various clinical trials in TNBC have explored the inhibition of a diverse range of RTKs and nRTKs, showing promising results. Inhibition strategies have encompassed EGF/EGFR targeted therapeutics, such as cetuximab, panitumumab, depatuxizumab, erlotinib, gefitinib, and liposomes carrying anti-EGFR fragments. Additionally, HER2 targeted therapeutics, including trastuzumab, NPS, and lapatinib, as well as VEGF/VEGFR targeted therapeutics like cediranib, apatinib, and bevacizimab, have been subjects of clinical testing. Furthermore, clinical trials have evaluated multi-RTK/nRTK targeted therapeutics, including sunitinib, sorafenib, pazopanib, cabozantinib, fortenib, and lenvatinib. These trials have demonstrated the potential of targeting this wide array of RTKs and nRTKs in TNBC treatment. RTK receptor tyrosine kinase, nRTK non-receptor tyrosine kinase, TNBC triple-negative breast cancer, EGFR epidermal growth factor receptor, VEGFR vascular endothelial growth factor receptor, NPS nelipepimut-S, RET rearranged during transfection, FGFR fibroblast growth factor receptor, FLT3 Fms related receptor tyrosine kinase 3, PDGF platelet-derived growth factor, PDGFR platelet-derived growth factor receptor, MET mesenchymal-epithelial transition factor, AXL anexelekto, RON récepteur d’origine nantais, TIE-2 tunica interna endothelial cell kinase 2, NPs-Ab nanoparticle bound antibody, EGF epidermal growth factor, VEGF vascular endothelial growth factor

**Fig. 4:**
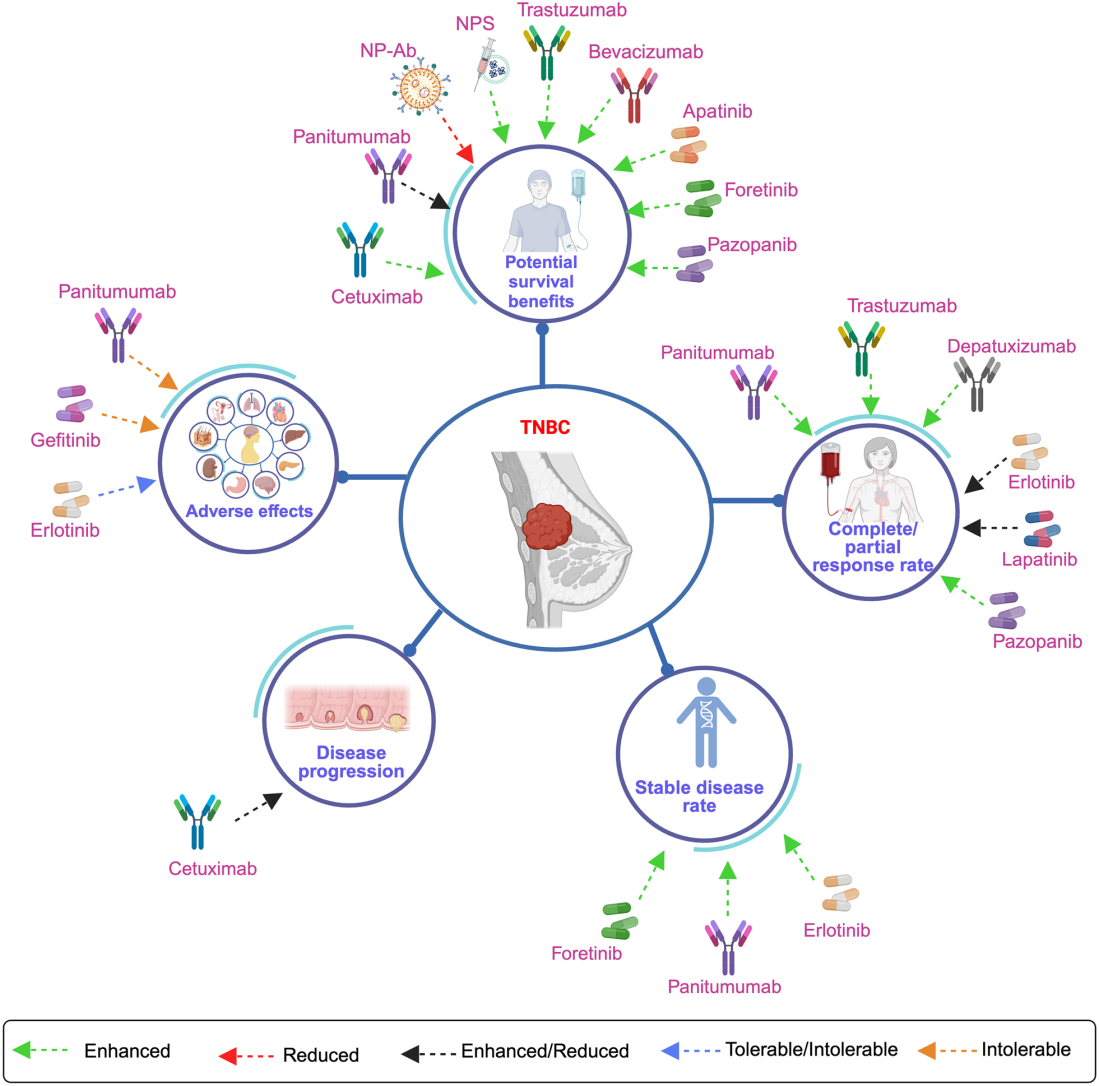
The RTK and nRTK targeted therapeutics have been shown to be efficacious in treating TNBC patients. Clinical trials across different phases have revealed varying outcomes upon the administration of TK targeted therapeuticsin combination with chemotherapeutic agents. Cetuximab, NPS, trastuzumab, bevacizumab, apatinib, fortenib, and pazopanib exhibit potential survival benefits in patients diagnosed with TNBC. Nevertheless, the substantiation of these benefits is predominantly reliant on findings from phase II clinical trials. However, the utilization of bevacizumab in conjunction with the chemotherapeutic agent paclitaxel has demonstrated an enhancement in progression-free survival rates, as evidenced by results from phase II clinical trials. It is noteworthy to highlight that the administration of bevacizumab with chemotherapy involving paclitaxel or other chemotherapeutic agents did not manifest potential survival benefits, as observed in phase III trials. This dichotomy shows the importance of discerning outcomes across different trial phases for a comprehensive understanding of the therapeutic efficacy and potential limitations of such treatment modalities. In addition, panitumumab failed to enhance patient survival due to intolerable adverse effects. Interestingly, panitumumab, trastuzumab, pazopanib, and depatuxizumab have been observed to improve the response rate. Conversely, treatment regimens involving erlotinib, lapatinib, and panitumumab have displayed mixed effects, indicating that these drugs might enhance the efficacy of certain chemotherapeutic agents selectively. RTK receptor tyrosine kinase, nRTK non-receptor tyrosine kinase, TNBC triple-negative breast cancer, TK tyrosine kinase, NP-Ab nanoparticle bound antibody, NPS nelipepimut-S

At present, the FDA has not sanctioned any TKIs specifically for addressing TNBC. However, a multitude of both published and ongoing clinical trials are dedicated to assessing the efficacy of these therapeutic agents, revealing encouraging outcomes among several TNBC patients. The prevailing emphasis of these trials resides in investigating compounds that target key molecular entities such as EGFR, VEGF/VEGFR, HER2, and PDGFR. Details of these clinical trials have been listed in Table 1 (published [48, 49, 57, 69, 90–133]) and Table 2 (registered under https://clinicaltrials.gov/). The time line, molecular targets and clinical outcome of these therapeutics have been illustrated in Figs. 2, 3 and 4 respectively.

**Table 1:** Published clinical trials of RTK/nRTK on Triple negative breast cancer

Target	Drug name (RTK/nRTK inhibitor)	Phase	No. of patients^#^	Outcome	Reference
AXL, MET, RET, VEGFR, HER2	Cabozantinib + trastuzumab	II	8	13% CBR; median PFS 2.4 months; 0% CNS ORR	[90]
AXL, MET, RON, TIE-2, VEGFR	Foretinib	II	45	46% CBR among evaluable patients	[91]
c-Kit, PDGFR, VEGFR	Pazopanib + paclitaxel + doxorubicin + cyclophosphamide	II	27	38% pCR; 46% cCR; IRFI 62.5%; 70% IRFI in patients with pCR; 57% IRFI in patients with non-pCR	[92]
c-Kit, PDGFR, VEGFR	Sorafenib + vinorelbine	I/II	17	No significant improvement in survival rate	[93]
c-Kit, CSFR, PDGFR, VEGFR, FLT3	Sunitinib + doxorubicin + paclitaxel + cyclophosphamide + G-CSF	II	30	27% pCR in breast, 23% pCR in breast and axilla; DFS not achieved	[94]
	Sunitinib	II	20	15% ORR	[95]
	Sunitinib	II	213	Median PFS 2 months, median OS 9.4 months; 3% ORR	[96]
EGFR	Anti-EGFR-ILs-dox	II	48	Anti-EGFR-ILs-dox is not recommended; 72.9% PD; median PFS 3.5 months	[97]
	Cetuximab + cisplatin	II	115	20% ORR ↑PFS (median PFS 3.7 months); OS (median OS 12.9 months)	[98]
	Cetuximab + taxane	I/II	18	Weekly therapy is feasible; median OS 12 months; median TTF 6 months	[99]
	Cetuximab + carboplatin	II	102	< 20% response; ↑OS (median OS 10.4 months)	[100]
	Depatuxizumab mafodotin	I/II	1	PR (patient with EGFR amplification)	[101]
	Erlotinib + bendamustine	I	11	Median PFS 3.7 months; 45% SD, 9% PR; 9% ORR; median OS 10.8 months; ↑lymphopenia; ↓CD4 counts	[102]
	Erlotinib + metformin	I	8	25% SD; median PFS 2 months	[103]
	Panitumumab + carboplatin + paclitaxel	II	14	46% ORR among evaluable patients	[104]
	Panitumumab + gemcitabine + carboplatin	II	71	42% ORR; median PFS 4.4 months; median OS 11.6 months	[105]
	Panitumumab + Nab-paclitaxel + carboplatin + fluorouracil + epirubicin + cyclophosphamide	II	19	42% pCR ↑sensitivity to chemotherapy	[106]
	gefitinib + epirubicin + cyclophosphamide	II	181 (TNBC + non-TNBC)	15% pCR in TNBC patients	[107]
EGFR, HER2	Lapatinib + paclitaxel	III	131	No benefit in TNBC	[48, 49]
ErbB family	Afatinib	II	29	Median PFS 7.4 weeks; 24.1% CBR	[108]
HER2	Trastuzumab + nelipepimut-S + GM-CSF	IIb	99	↑Overall 36-month DFS (84.5%); 36-month DFS of patients with HER2 IHC 1^+^ (94.1%); 36-month DFS of patients with HLA-A24^+^ (96.2%); 36-month DFS of patients received NCT (77.0%)	[109]
	Trastuzumab + nelipepimut-S + GM-CSF	IIb	97	↑24-months DFS (92.6%)	[110]
	Trastuzumab + epirubicin + cyclophosphamide + 5-fluorouracil + cisplatin + docetaxel	II	80 (TNBC + non-TNBC)	36% pCR	[111]
MET	Tivantinib	II	22	5% ORR, 5% 6-month PFS	[112]
VEGF	Bevacizumab + epirubicin + cyclophosphamide + docetaxel	III	663	↑pCR (61.5% in *BRCA1/2* mutation^+^; 35.6% in *BRCA1/2* mutation^−^)	[113]
	Bevacizumab + taxane + gemcitabine + capecitabine or vinorelbine	III	159	41% ORR; ↑PFS (median PFS 6 months); OS (median OS 17.9 months)	[114]
	Bevacizumab + epirubicin + cyclophosphamide + docetaxel	III	663	↑pCR (39.3%)	[115]
	Bevacizumab + epirubicin + cyclophosphamide + docetaxel	III	663	No improvement in DFS & OS; pCR (11.4%)	[116]
	Bevacizumab + gemcitabine	II	19	Median PFS 3.9 months; median OS 16.1 months	[117]
	Bevacizumab + nivolumab + paclitaxel	II	17	59% ORR; 94% DC; 59% PR; 35% SD	[118]
	Bevacizumab + paclitaxel	II	38	Median PFS 9.6 months	[119]
	Bevacizumab + paclitaxel	III	100	No significant improvement of OS; inferior PFS	[57]
	Bevacizumab + docetaxel + epirubicin	I/II	10	66.3% ORR; 15.7% CR; 50.6% PR; ↓CTCs; Potentially toxic^*^	[120]
	Bevacizumab + durvalumab	Ib	9	Pre-treatment with bevacizumab makes patients more prone to benefit from durvalumab; Median OS 7.4 months; 44% CBR	[121]
	Bevacizumab + paclitaxel/capecitabine	III	130	Non-significant OS & PFS trends	[122, 123]
	Bevacizumab + paclitaxel	II	42	42.9% pCR; overall DFS 87.1%^*^; safe	[124]
	Bevacizumab + Nab-paclitaxel + carboplatin	II	12	50% pCR	[125]
	Bevacizumab + Nab-paclitaxel + gemcitabine	II	13	84.6% CBR; 82.5% OS; 38.4% CR; 30.7% PR; 6.9% SD; 6.9% PD; 10.6% PFS	[126]
	Bevacizumab + anthracycline/taxane	III	2591	No difference in OS	[127]
VEGFR	Cediranib + olaparib	I	8	No significant clinical activity; median PFS 3.7 months	[128]
VEGFR-2	Apatinib	IIa	25	Median PFS 4.6 months; median OS 8.3 months	[69]
	Apatinib	IIb	59	Median PFS 3.3 months; median OS 10.6 months; 10.7% ORR; 25% CBR	[69]
	Apatinib + camrelizumab + fuzuloparib	Ib	32	6.9% ORR; median PFS 5.2 months; 64.2% 12-month OS; 62.1% DC	[129]
	Apatinib + docetaxel + epirubicin + cyclophosphamide	II	31	54.8% pCR, 93.5% ORR, 100% DC; manageable toxicities; 90.9% EFS; 94.4% OS; tumor shrinkage in 96.8% patients	[130]
	Apatinib + SHR-1210 (anti-PD-1 antibody)	II	12	Median PFS 3.7 months ↑tumor-infiltrating CD8^+^ T-cells; CD19^+^ B-cells; OPN; TGF-β	[131]
	Ramucirumab + eribulin	II	43	Larger benefit observed in TNBC subgroup	[132]
WEE-1	Adavosertib + cisplatin	II	34	26% ORR; median PFS 4.9 months; ↑memory CD4^+^ T cells; anti-tumor M1 macrophages	[133]

*Anti-EGFR-ILs-dox* anti-egfr immunoliposomes loaded with doxorubicin, *AXL* anexelekto, *BRCA* breast cancer gene, *CBR* clinical benefit rate, *cCR* clinical complete response, *CD* clusters of differentiation, *CNS* central nervous system, *CR* complete response, *CSFR* colony stimulating factor receptor, *CTC* circulating tumor cell, *DC* disease control, *DFS* disease-free survival, *EFS* event-free survival, *EGFR* epidermal growth factor receptor, *ErbB* erythroblastic leukemia viral oncogene homologue, *FLT3* Fms related receptor tyrosine kinase 3*, GM-CSF* Granulocyte macrophage-colony stimulating factor, *HER2* human epidermal growth factor receptor 2, *HLA-A24* human leukocyte antigen-A24, *IHC* immunohistochemistry, *IRFI* invasive recurrence-free interval, *MET* mesenchymal-epithelial transition factor, *Nab-paclitaxel* nanoparticle albumin bound *paclitaxel*, *NCT* neoadjuvant chemotherapy, *nRTK* non-receptor tyrosine kinases, *OPN* osteopontin, *ORR* objective response rate, *OS* overall survival, *pCR* pathological complete response, *PD* progressive disease, *PD-1* programmed cell death protein 1, *PDGFR* platelet derived growth factor receptor, *PFS* progression-free survival, *RTK* receptor tyrosine kinases, *RET* rearranged during transfection*, RON* récepteur d’origine nantais, *SD* stable disease, *TGF-β* transforming growth factor-β, *TIE-2* tunica interna endothelial cell kinase 2, *TK* tyrosine kinases, *TNBC* triple negative breast cancer, *TTF* time to treatment failure, *VEGF* vascular endothelial growth factor, *VEGFR* vascular endothelial growth factor receptor

*These results are for all the patients including TNBC; ^#^Number of TNBC patients only

**Table 2:** Clinical trials registered at (https://clinicaltrials.gov/) on RTKs/nRTKs in TNBC as accessed on 13th March 2024

RTK/nRTK	Drug	Phase	No. of patients	Place/institute	Start date	ID
AXL, c-Kit, MERTK, RET, TYRO3, VEGFR-2	Sitravatinib + tislelizumab ± Nab-paclitaxel	II	98	Fudan University, Shanghai, China	2021–04	NCT04734262
BCR-ABL, c-Kit, EPHA2, PDGFR-β, SRC family (FYN, LCK, SRC, YES)	Dasatinib^*^	II	22	Baylor College of Medicine, Houston, Texas, United States	2008–12	NCT00817531
	Dasatinib^*^ + icosapent ethyl	I/II	1	University of Texas MD Anderson Cancer Center LAO, Houston, Texas, United States	2022–11	NCT05198843
Bruton’s tyrosine kinase	Ibrutinib + durvalumab	Ib/II	124	Pharmacyclics LLC, United states	2015–03	NCT02403271
c-Kit, FGFR, PDGFR, VEGFR	Anlotinib + TQB2450	III	332	Chia Tai Tianqing Pharmaceutical Group Co., Ltd., China	2020–06	NCT04405505
EGFR	Gefitinib	II	50	Jiangmen Central Hospital, Jiangmen, China	2013–01	NCT01732276
	Nimotuzumab ± docetaxel + capecitabine	II	90	Chinese Academy of Medical Sciences, China	2013–09	NCT01939054
FGFR, c-Kit, PDGFR-α, RET, VEGFR	Lenvatinib ± pembrolizumab	II	590	Merck Sharp & Dohme LLC	2019–02	NCT03797326
FGFR-1, -2, -3, PDGFR, VEGFR	Lucitanib^*^	II	178	Clovis Oncology, Inc., United States	2014–09	NCT02202746
MET, VEGFR-2	Cabozantinib	II	35	Dana-Farber Cancer Institute, Boston, United States	2013–02	NCT01738438
VEGF, VEGFR-2	Apatinib mesylate + albumin-bound paclitaxel/bevacizumab + albumin-bound paclitaxel	II	128	First Affiliated Hospital of Bengbu Medical College, Bengbu, China	2022–01	NCT05192798
VEGFR	Fruquintinib	I	129	HUTCHMED International	2017–11	NCT03251378
VEGFR-2	Apatinib + camrelizumab	II	58	Sun Yat-Sen Memorial Hospital of Sun Yat-Sen University, China	2022–12	NCT05556200
WEE-1	AZD1775 + cisplatin	II	34	Dana-Farber Cancer Institute, Boston, United States	2017–01	NCT03012477

*ABL* abelson, AXL anexelekto, *BCR* break point cluster, *EGFR* epidermal growth factor receptor, *EPHA2* ephrin type-A receptor 2, *FGFR* fibroblast growth factor receptor, *FYN* FYN protooncogene, *LCK* lymphocyte-specific protein tyrosine kinase, *MERTK* Mer receptor tyrosine kinase 3, *MET* mesenchymal-epithelial transition factor, *Nab-paclitaxel* nanoparticle albumin bound *paclitaxel*, *nRTK* non-receptor tyrosine kinases, *PDGFR* platelet derived growth factor receptor, RET rearranged during transfection, *RTK* receptor tyrosine kinases, *SRC* SRC protooncogene*, TNBC* triple negative breast cancer, *VEGF* vascular endothelial growth factor, *VEGFR* vascular endothelial growth factor receptor, *TYRO3* tyrosine kinase receptor 3*, YES*, Yamaguchi sarcoma oncogene

*Terminated

### Erythroblastic leukemia viral oncogene homologue (ErbB) family targeted therapeutics

The ErbB family of receptors represents the subset within the broader RTK superfamily, encompassing 4 distinct members: EGFR (ErbB1), HER2 (ErbB2), ErbB3, and ErbB4. Notably, EGFR and HER2 have been recognized for their aberrant activation patterns in diverse human malignancies [134, 135]. Several TK targeted therapeutics have been developed to target constituents of this receptor family, finding application in the management of various carcinoma types [135]. In the pursuit of therapeutic advancements, clinical trials have been undertaken to assess the effectiveness and tolerability of these targeted therapeutics in the context of TNBC. For instance, afatinib (BIBW 2992), an orally bioavailable small molecule inhibitor with activity against all members of the ErbB family, was subjected to evaluation in extensively pre-treated HER2-negative breast cancer patients through a phase II investigation. Nevertheless, the outcomes of this study proved to possess modest efficacy, as a mere 24.1% of TNBC patients derived clinical benefit, with only 10.3% experiencing sustained disease stability at least a span of 4 months [108]. In the AE37 cohort of the clinical trial, discernible advantages were observed for individuals presenting advanced-stage malignancy, HER2 under-expression, and TNBC upon administration of the AE37 vaccination along with GM-CSF. Notably, subjects exhibiting both advanced-stage disease and HER2 under-expression exhibited a marked clinical benefit in response to AE37 vaccination in combination with GM-CSF. This was exemplified by an earlier attainment of a disease-free survival (DFS) plateau, a status sustained over the 10-year duration of post-intervention follow-up [136].

#### EGFR targeted therapeutics

The EGFR, belongings to the ErbB family of RTKs, is prominently identified as overexpressed in more than 50% of TNBC cases [67]. Given its pivotal role in eliciting various downstream signaling cascades crucial for cell proliferation, cell cycle progression, cell survival, and tumor growth, EGFR represents a prominent target for therapeutic intervention [50, 67]. A diversity of EGFR targeted therapeutics have been developed and have garnered clinical attention in diverse cancer types, including investigations within the TNBC domain [135]. To this end, clinical trials have sought to evaluate the effectiveness and tolerability of EGFR targeted therapeutics within the TNBC context. For instance, a phase I/II trial investigated the combination of weekly paclitaxel therapy with cetuximab, a chimeric anti-human EGFR antibody, on TNBC patients. This trial exhibited prolonged toleration of the combination regimen and yielded a median survival period of 12 months [99]. Additionally, a randomized phase II study in TNBC patients indicated that cetuximab might enhance the activity of chemotherapy when administered alongside taxanes such as paclitaxel or docetaxel, yielding a median survival of 12 months and a time to treatment failure of 6 months [99]. Another phase II investigation probed the combined use of cetuximab with cisplatin, revealing substantially prolonged median PFS and OS compared to cisplatin therapy alone. These findings warrant further exploration in the metastatic TNBC [98]. Nevertheless, the TBCRC 001 study, a two-arm phase II assessment of cetuximab with or without carboplatin, revealed that although the combination correlated with a slightly superior median OS, neither arm demonstrated a decrease in disease progression [100].

In parallel, an alternate fully human immunoglobulin G (IgG)2 mAb, panitumumab, combined with neoadjuvant chemotherapy (NCT), exhibited considerable efficacy in a single-arm phase II study involving HER2-negative inflammatory breast cancer patients. Notably, this study recorded a remarkable pathological complete response (pCR) rate (42%) in these patients, suggesting a potential augmentation of chemotherapy sensitivity [106]. However, the assessment of panitumumab in conjunction with paclitaxel and carboplatin together in a phase II trial for metastatic TNBC reported a 46% overall response rate but indicated challenges in tolerability compared to alternative regimens [104]. Likewise, an analogous phase II study combining panitumumab with a standard gemcitabine and carboplatin regimen in metastatic TNBC unveiled a 42% response rate but fell short of achieving the estimated PFS, reporting a median of 4.4 months. Intriguingly, the median OS was noted to be 11.6 months, which is similar to patients treated solely with chemotherapy, highlighting the challenge of enhancing therapeutic outcomes [105]. Notably, depatuxizumab mafodotin, an anti-EGFR mAb linked to a cytotoxin, exhibited partial responses in EGFR-amplified TNBC patients during a phase I/II study involving advanced solid tumors with a propensity for EGFR overexpression [101]. Additionally, small molecule inhibitors targeting EGFR are under exploration. For example, a phase I study investigated the combination of the EGFR inhibitor erlotinib with the biguanide antidiabetic agent metformin at varying doses. Although the combination exhibited tolerable adverse event profiles, the clinical outcomes remained inadequate [103]. Conversely, a phase I study investigating erlotinib in combination with bendamustine, a distinct chemotherapeutic hybrid compound, among stage IV metastatic TNBC patients, reported intolerable levels of adverse events, despite a 45% achievement of stable disease [102]. Similarly, a phase II study integrating gefitinib, an anti-EGFR agent, with the epirubicin and cyclophosphamide combination, exhibited higher incidences of adverse events and did not yield the anticipated increase in pCR rate. Nonetheless, the rate of pCR among TNBC patients was greater compared to non-TNBC patients [107]. The SAKK 24/14 study, a phase II exploration of an anti-EGFR targeted nanocontainer drug incorporating anti-EGFR antibody fragments into PEGylated liposome for patients with advanced TNBC, resulted in notably poor median PFS, failing to meet the primary endpoint [97]. Further, a number of clinical trials (registered in https://clinicaltrials.gov/) continue to examine EGFR TKIs within the realm of TNBC treatment. One such phase II trial aims to assess the clinical benefit rate (CBR), PFS, and toxicity of gefitinib in breast cancer patients (NCT01732276). Another ongoing phase II investigation endeavors to measure the objective response rate (ORR), PFS, and relationship of serum/tissue EGFR after the treatment of docetaxel and capecitabine in conjunction with nimotuzumab, an IgG1 humanized mAb recognizing an epitope on the extracellular domain of EGFR (NCT01939054).

#### ErbB2 targeted therapeutics

ErbB2, alternatively referred to as HER2, represents a transmembrane glycoprotein belonging to the ErbB family of proteins, distinctly acknowledged as an oncogene commonly overexpressed in breast cancer [18, 137]. Targeted therapy directed at HER2 has displayed limited efficacy among patients exhibiting low levels of the proteins. However, investigational efforts encompassing combinatory therapy involving HER2 targeted therapeutics are currently underway and offer potential promise for the management of TNBC [109]. For instance, a mAb targeting this receptor, trastuzumab, in conjunction with granulocyte–macrophage colony-stimulating factor (GM-CSF), either with or without nelipepimut-S (NPS), a HER2-derived peptide vaccine, was evaluated within a randomized, multicenter, single-blinded, phase IIb trial encompassing high-risk breast cancer participants characterized by low levels of HER2 expression, including TNBC cases. Notably, outcomes revealed substantial enhancements in the DFS rate, escalating from 70.2 to 92.6% in the NPS-receiving group, thereby proposing a synergistic interaction in the combination [110]. Analogously, a phase IIb investigation by Chick et al. [109] yielded similar findings, wherein TNBC patients significantly benefited from this vaccine, contributing to an elevated DFS rate (84.5% vs. 70.6%). In-depth subgroup analysis of TNBC patients highlighted those individuals classified as human leukocyte antigen (HLA)-A24^+^ and HER2^+^ immunohistochemistry (IHC) expression, alongside those who had undergone prior NCT, experienced substantial increments in DFS. In a multicenter, phase II study employing the combination of trastuzumab with NCT comprising FEC100 (epirubicin, cyclophosphamide, and 5-fluorouracil), succeeded by cisplatin–docetaxel, a noteworthy 36% of TNBC patients achieved pCR [111]. Conversely, the utilization of lapatinib, a dual inhibitor targeting both EGFR and HER2, alongside paclitaxel therapy, did not yield beneficial outcomes in TNBC patients [48].

#### VEGF/VEGFR targeted therapeutics

Angiogenesis denotes the intricate process of generating new blood vessels and a supporting matrix from preexisting capillaries, ultimately forming a mature vascular network. This phenomenon is intricately governed by a balance of pro- and anti-angiogenic factors [138, 139]. Among the pivotal contributors to angiogenesis are the members of the VEGF family, along with their corresponding transmembrane RTKs VEGFR-1, -2, and -3 [139, 140]. These molecular components play an instrumental role in fostering neo-angiogenesis and augmenting vascular permeability within tumors [66, 72]. The overexpression of this protein family has been linked to diminished OS, reduced relapse-free survival, and compromised responsiveness to anti-cancer interventions in TNBC [66, 139].

Recent years have witnessed a plethora of clinical investigations demonstrating the effectiveness of TK targeted therapeutics for VEGF/VEGFR pathway. A prominent exemplar is bevacizumab, a humanized mAb that specifically targets VEGF-A, thereby impeding its interaction with VEGFR. This agent has undergone assessments both as a monotherapy and in conjunction with chemotherapeutic agents among TNBC patients [138, 140, 141]. Notably, a phase II study employing nanoparticle albumin-bound paclitaxel (Nab-paclitaxel) alongside bevacizumab and gemcitabine showcased a remarkable CBR of 86.4%, accompanied by 38.4% complete responses, 30.7% partial responses, and an OS rate of 82.5%. This regimen yielded a low occurrence of adverse events while demonstrating efficacy [126]. Similarly, a regimen featuring biweekly administration of bevacizumab and weekly Nab-paclitaxel in conjunction with carboplatin achieved a substantial pathological pCR rate of 50% among TNBC patients, signifying its therapeutic value [125]. Furthermore, the combination of bevacizumab with liposomal doxorubicin, paclitaxel, and cyclophosphamide showcased favorable safety profiles and notable efficacy in a phase II study, indicating its potential utility [124].

A noteworthy exploration encompassed the combination of bevacizumab with an anti-PD-L1 mAb, durvalumab, in patients with advanced HER2 negative breast cancer. This intervention engendered CBR of 44% after 4 months, alongside enhanced immune signatures, alluding to an immune-priming effect of bevacizumab [121]. Additionally, a phase II study examined the combination of bevacizumab with weekly paclitaxel among Japanese patients with metastatic breast cancer and showed median PFS of 9.6 months among patients with locally recurrent or metastatic TNBC [119]. The addition of bevacizumab to diverse chemotherapeutic regimens, encompassing taxane, gemcitabine, capecitabine, or vinorelbine, revealed significant enhancements in PFS and a pronounced benefit in TNBC patients in a randomized phase III trial [114]. Such combinations demonstrated particular promise among patients carrying *BRCA1/2* mutations, indicating potential stratification strategies [113]. Interestingly, bevacizumab in combination with nivolumab and paclitaxel showed ORR of 59% in TNBC patients [118]. Nevertheless, certain clinical endeavors, such as the BEATRICE study, have not yielded substantial improvements in early TNBC patients, and the integration of bevacizumab with chemotherapy and other inhibitors has shown mixed outcomes [57, 115–117, 120, 127]. To this end, while bevacizumab holds promise in inhibiting TNBC progression, further investigations are essential to ameliorate adverse events and optimize therapeutic combinations.

A distinct TKI, apatinib, has garnered attention as a second-generation inhibitor targeting phosphorylation of the intracellular domain of VEGFR-2. Trials assessing apatinib in metastatic TNBC have indicated promising results, showcasing encouraging PFS rates and manageable toxicities [69, 129, 131]. Moreover, apatinib has shown synergy with other therapeutic agents, yielding positive outcomes in terms of PFS, disease control rate (DCR), and immune cell infiltration [130, 131]. Numerous ongoing studies continue to explore apatinib’s potential as a treatment avenue for TNBC patients. Combination therapies involving eribulin and ramucirumab have demonstrated promise in the TNBC subgroup of certain trials, yet have not consistently shown significant improvement in PFS compared to monotherapy [132]. Likewise, the combination of cediranib and olaparib exhibited manageable adverse events, but yielded limited clinical benefits for TNBC patients [128]. Clinical trials have also shown that combination therapy of bevacizumab along with paclitaxel/capecitabine did not show significant improvement in OS and PFS trends [122, 123]. Ongoing clinical investigations continue to unravel the potential of these agents in the TNBC landscape.

In sum, the VEGF/VEGFR pathway emerges as a compelling target for impeding TNBC progression, though extensive phase III clinical studies are needed to establish the efficacy of existing agents and to unearth novel compounds with enhanced inhibitory potential.

### Multi-TKIs

Numerous compounds have been subjected to clinical trials, targeting diverse TKs, with the potential to yield several advantages, including attenuated prospects for resistance emergence and increased precision in addressing distinct pathways implicated in the commencement and advancement of TNBC [94, 142, 143]. A phase II multicenter trial examined foretinib efficacy, wherein, an oral agent functioning as a multi-kinase inhibitor against MET, RON, AXL, angiopoietin-1 receptor [TEK or tunica interna endothelial cell kinase 2 (TIE-2)], and VEGFR, was employed. This trial engaged patients diagnosed with locally recurrent or metastatic TNBC, revealing a CBR of 46%. Notably, 40.5% of subjects achieved stable disease, extending for a median duration of 5.4 months. The outcome prompts further evaluation of foretinib as a potential front-line chemotherapy for metastatic TNBC [91]. In addition, a single-arm, phase II study focused on investigating the combination of pazopanib, an oral selective small molecule targeting an array of RTKs encompassing VEGFR-1, -2 and -3, PDGFR-α and -β, and c-Kit, in conjunction with a weekly paclitaxel regimen. This endeavor yielded a pCR rate of 38% among TNBC patients, accompanied by a 46% clinical complete response (cCR) rate. Nonetheless, the 2-year invasive recurrence-free interval (IRFI) was merely 70% for pCR patients, as opposed to 57% in those devoid of pCR status [92]. Similarly, a multicenter, open-label phase II trial aimed to ascertain the anti-tumor activity of sunitinib malate, an oral multi-TKI targeting colony stimulating factor receptor (CSFR), VEGFR-1, -2, and -3, FMS related receptor tyrosine kinase 3 (FLT3), c-Kit, PDGFR-α, and -β, among patients with advanced solid metastatic breast tumors who had previously undergone anthracycline and taxane treatment. This investigation reported a 15% response rate in TNBC patients, signifying sunitinib’s activity [95]. Nevertheless, sunitinib’s efficacy reduced when used as monotherapy for advanced TNBC, with a randomized phase II study disclosing a median PFS of 2 months, notably lower than the standard of care arm, which reported a PFS of 2.7 months. Further, sunitinib did not extend OS, leading to the exclusion of its recommendation for TNBC treatment [96]. Correspondingly, a phase II study exploring the efficacy of cabozantinib, a small molecule, multi-target inhibitor targeting MET, AXL, rearranged during transfection (RET), HER2, and VEGFR, either alone or with trastuzumab, in heavily pretreated breast cancer patients with brain metastasis, yielded only a 13% CBR and a median PFS of 2.4 months for TNBC patients [90]. Another phase II study assessing cabozantinib on metastatic TNBC reported a 9% ORR, a 2-month median PFS, and a 34% CBR (NCT01738438). Likewise, sorafenib, an oral multi-TKI targeting VEGFR, PDGFR, FLT3, and c-Kit, was combined with vinorelbine in a phase I/II trial for metastatic breast cancer, including TNBC. However, no significant advantage was observed when compared to vinorelbine monotherapy, though its potential in treatment-naive patients remains of interest [93].

Ongoing clinical trials continue to assess multi-TKIs for TNBC treatment, holding substantial promise for transforming therapeutic strategies. For instance, a phase II trial investigates the combination of sitravatinib, a broad-spectrum small molecule inhibitor targeting AXL, c-Kit, Mer receptor tyrosine kinase 3 (MERTK), RET, TYRO3, and VEGFR-2 alongside tislelizumab, a PD-1 specific humanized IgG4 mAb, with different dosages, in conjunction with or without Nab-paclitaxel, for recurrent or metastatic TNBC. Parameters such as ORR, PFS, OS, DCR, potential outcome-associated biomarkers, and adverse events were aimed to be analysed (NCT04734262). Another parallel study, a multicenter open-label phase II endeavor, aims to gauge the safety and efficacy of the combination therapy involving lenvatinib, a small-molecule TKI inhibiting VEGFR-1, -2, -3, FGFR-, -2, -3, -4, PDGFR-α, c-Kit, and RET, alongside pembrolizumab, targeting PD-1, among previously treated patients with solid tumors, including TNBC (NCT03797326). Additionally, an open-label multicenter phase II study assessing lucitanib, an orally administered multi-TKI binding to FGFR-1, -2, -3, VEGFR-1, -2, -3, and PDGFR-α and -β, displayed median PFS of 93 d and 77 d, along with ORR of 48.1% and 34.3% for 10 mg and 15 mg administered groups, respectively (NCT02202746). Although terminated, a neoadjuvant phase II trial of estrogen receptor (ER)^−^ breast cancers, including TNBC, appraised the biologic effectiveness of dasatinib, a multi-targeted TKI targeting the Src family, breakpoint cluster region-abelson (BCR-ABL), PDGFR-β, and c-Kit, with interim analysis indicating stable disease in 15 out of 22 patients (NCT00817531). This compound is being further explored in combination with icosapent ethyl, an omega-3 fatty acid acting as a lipid-regulating agent, for metastatic TNBC (NCT05198843). Furthermore, anlotinib hydrochloride, a multi-TKI targeting VEGFR, FGFR, PDGFR, and c-Kit, is currently under a randomized phase III study, combined with TQB2450 injection, in TNBC patients. This study assesses PFS, ORR, and DCR, compared with albumin-bound paclitaxel injection (NCT04405505).

In conclusion, multi-TKI compounds hold great potential for revolutionizing the TNBC treatment landscape, despite the current scarcity of efficacious drugs. Future research endeavors are essential for refining therapeutic approaches and meeting the unmet demand for effective, precise, and low-toxicity treatments.

### Other promising targets

Numerous unexplored RTKs and nRTKs have been implicated in the context of TNBC. The dysregulated expression of the RTK and MET has been identified as a driving force behind tumorigenesis, contributing to unfavorable prognosis and augmented resistance in various cancers, including TNBC [144–146]. To illustrate, a single-arm, phase II investigation conducted by Tolaney et al. [112] assessed the safety and effectiveness of tivantinib, an oral small molecule inhibitor targeting MET, as monotherapy in metastatic TNBC. The study yielded poor outcomes, with notably low overall response rate and median PFS, where only a single patient exhibited partial response to the treatment. WEE1, classified as a TK that governs inhibition of cyclin dependent kinase (CDK)1 and CDK2, thereby serving as a negative regulator of the cell cycle, holds relevance in this context [147, 148]. An analysis involving patients with metastatic breast cancer showed the efficacy and molecular responses to adavosertib (AZD1775), a WEE1 inhibitor, combined with cisplatin therapy. However, the study failed to attain the predefined ORR threshold of > 30%, as it reported an ORR of 26%. The median PFS was measured at 4.9 months, and notable increase in memory CD4^+^ T cells were observed in these patients [133]. An ongoing phase II clinical trial seeks to assess the safety and efficacy of the same combination therapy (cisplatin plus AZD1775) in metastatic TNBC. In addition to evaluating median PFS and ORR, the study will gauge alterations in phosphorylated cell division cycle 2 (pCDC2) following therapy, the presence of *p53* and *BRCA1/2* mutations, and potential correlations between next-generation sequencing of tumors and participant outcomes (NCT03012477). Bruton’s TK (BTK), a non-receptor kinase, has been linked to oncogenic signaling pathways that contribute to enhanced cell survival and proliferation [149]. While the investigation of BTK inhibitors in the context of TNBC remains limited, a multicenter phase Ib/II study involving patients with relapsed or refractory solid tumors, including TNBC, has been designed to assess the tolerability and efficacy of combining a BTK inhibitor, ibrutinib, with durvalumab (MEDI4736; NCT02403271).

In summary, our review survey encompassed 46 clinical trials involving various TK inhibitors in TNBC patients as obtained from PubMed. These trials comprised 5 phase I, 29 phase II, 4 phase I/II, and 8 phase III studies. Additionally, 14 clinical trials were registered on https://clinicaltrials.gov/, of which 3 were terminated prematurely. The distribution of clinical trials, both published and unpublished, targeting specific TKs is presented in Table 3. Results from these trials revealed heterogeneous responses among TNBC patients. Noteworthy, certain trials exhibited promising outcomes, advocating for their progression to subsequent phases for the benefit of TNBC patients. For instance, foretinib, a multi-TK inhibitor, demonstrated a CBR of 46% in metastatic TNBC patients, prompting consideration for phase III trials [91]. Additionally, anti-EGFR therapies such as cetuximab with taxane, cetuximab with carboplatin, and depatuxizumab mafodotin were evaluated for their efficacy in patients with EGFR overexpression, warranting further investigation [99–101]. Notably, cetuximab in combination with cisplatin demonstrated improved ORR and appeared to extend PFS and OS in TNBC patients, suggesting the need for large-scale clinical trials [98]. Moreover, panitumumab displayed varying effects when combined with different chemotherapeutic agents, suggesting potential benefits with careful selection of chemotherapeutic partners [104–106]. Treatment with the HER2 antibody trastuzumab demonstrated benefits for TNBC patients, as indicated by enhanced response rates, warranting phase III evaluation [109–111]. Further, preliminary data on the VEGFR-2 inhibitor apatinib showed anti-tumor activity and efficacy of chemotherapeutic agents, necessitating further validation [69, 129, 131]. In a separate clinical trial, the combination of sorafenib with vinorelbine did not yield a significant enhancement in patient survival rates compared to the historical data associated with vinorelbine alone. This outcome suggests the necessity for further assessment of efficacy of sorafenib in TNBC patients who have not been treated with bevacizumab, albeit only if a specific cohort of patients is delineated [93]. Hence, we advocate for meticulous patient cohort selection and judicious choice of TK inhibitors in subsequent phase trials involving the TNBC population. Conversely, some inhibitors or mAbs exhibited increased toxicity or lack of efficacy, rendering their advancement impractical. For instance, the multi-TK inhibitor cabozantinib, when combined with trastuzumab, demonstrated tolerability and efficacy for anti-vascular effects but lacked efficacy as an anti-tumor agent in TNBC patients during phase II trials [90]. Another clinical trial showed that pazopanib in combination with chemotherapy resulted in high discontinuation rates due to hematological toxicity and failed to demonstrate apparent benefits, suggesting no further investigation is warranted in the TNBC population [92]. Sunitinib therapy, alone or in combination with chemotherapeutic agents, did not significantly benefit TNBC cohorts, with no improvement in response rates [94–96]. Another study showed anti-EGFR-ILs-dox should not be further developed for TNBC [97]. Similarly, erlotinib, an EGFR inhibitor, in combination with bendamustine was deemed unfeasible due to an unacceptable rate of lymphopenia occurrence leading to life-threatening infections [102]. Bevacizumab, a promising mAb, demonstrated efficacy in phase II trials but failed to replicate these results in phase III trials [57, 113–127]. Hence, meticulous evaluation of these RTK/nRTK inhibitors/mAbs as monotherapy or in combination with other agents is warranted, along with appropriate patient stratification based on genetic profiles.

**Table 3:** Summary of clinical trials conducted on each RTKs/nRTKs

Targeted RTK/nRTK	No. of published clinical trials (*n* = 46)				No. of unpublished clinical trials (*n* = 14)			
	Phase I	Phase I/II	Phase II	Phase III	Phase I	Phase I/II	Phase II	Phase III
Bruton’s TK	–	–	–	–	–	1	–	–
EGFR	2	2	7	–	–	–	2	–
EGFR, HER2	–	–	–	1	–	–	–	–
ErBb family	–	–	1	–	–	–	–	–
HER2	–	–	3	–	–	–	–	–
MET	–	–	1	–	–	–	–	–
MET, VEGFR-2	–	–	–	–	–	–	1	–
VEGF	1	1	6	7	–	–	–	–
VEGF, VEGFR-2	–	–	–	–	–	–	1	–
VEGFR, VEGFR-2	2	–	4	–	1	–	1	–
WEE-1	–	–	1	–	–	–	1	–
Multi-TKs	–	1	6	–	–	1	4	1

Data about unpublished clinical trials are retrieved from https://clinicaltrials.gov/

“–” indicate no data

*EGFR* epidermal growth factor receptor, *ErbB* erythroblastic leukemia viral oncogene homologue, *HER2* human epidermal growth factor receptor 2, *MET* mesenchymal-epithelial transition factor, *nRTK* non-receptor tyrosine kinases, *RTK* receptor tyrosine kinases, *TK* tyrosine kinases, *VEGF* vascular endothelial growth factor, *VEGFR* vascular endothelial growth factor receptor

## Current limitations and future prospective

It is crucial to acknowledge that the exclusive inhibition of TK, whether employing standalone inhibitors or in conjunction with chemotherapeutic agents, has thus far failed to achieve total remission in cases of breast carcinomas. The RTK and nRTK inhibitors that exhibited promising benefits in early-phase clinical trials (phase I and II) have not been successful in demonstrating enhanced clinical advantages concerning response rates and/or survival rates in the large cohorts of TNBCs during phase III trials. This limitation is attributed to various challenges, including the acquired treatment resistance, severe drug toxicity, off-target effects and suboptimal efficacy [39, 41, 150]. Furthermore, the presence of carboxyterminal fragments of HER2, specifically p95HER2, and the occurrence of exon skipping splice variant HER2D16, have been documented in association with the development of resistance to osimertinib in NSCLC and trastuzumab in breast cancer, respectively [151–153]. Additionally, the resistance exhibited by TNBC to EGFR inhibition may be attributed to the dimerization of EGFR with the AXL, thereby bypassing the inhibitory effect on EGFR and sustaining the activation of the mTOR in TNBC cells [154]. Instead of targeting EGFR, the blockade of AXL utilizing 20G7-D9 mAb demonstrated efficacy in restraining tumor growth and metastasis in a patient-derived TNBC xenograft model [155]. Despite the encouraging preclinical outcomes, a phase II trial involving the AXL inhibitor (bemcentinib) was terminated because there were no instances of complete or partial responses observed in advanced TNBC patients (NCT03184558). This delineates the current gaps in our understanding of the genetic diversity and molecular mechanisms associated with RTKs and nRTKs, necessitating further investigations. Conversely, the ubiquitous expression of EGFR in TNBC tumor has spurred recent investigations towards the development of EGFR-targeted ADCs, exemplified by aminoflavone-loaded anti-EGFR unimolecular micelle nanoparticles and ABT-414, anti-EGFR antibody conjugated to cytotoxic monomethyl auristatin F [156–158]. These ADCs demonstrated commendable anti-tumor efficacy in murine models harboring EGFR-positive TNBC tumors [156–158]. Nevertheless, comprehensive evaluation through diverse phases of clinical trials is imperative to definitively establish the potential therapeutic benefits of these conjugates for patients.

An additional and innovative resistance mechanism to TKs has emerged from observations in studies investigating VEGFR blockers. It is pertinent to highlight that antiangiogenic agents, including sunitinib, have been observed to induce hypoxia and augment the population of cancer stem cells in TNBC in vivo [159]. Nonetheless, numerous VEGF signaling inhibitors are undergoing clinical trials for advanced TNBC treatment in conjunction with chemotherapeutic and/or immunotherapeutic agents (Table 2). However, the withdrawal of approval for bevacizumab in metastatic breast cancer stemmed from safety concerns outweighing survival benefits, generating controversies and potentially diminishing confidence in employing VEGFR blockers in TNBC patients [160, 161]. The clinical ramifications of this phenomenon necessitate further evaluation.

The development of novel TK targeted therapeutics employing diverse mechanisms to target specific entities with minimal toxicity is an ongoing research pursuit. An alternative avenue to overcome this indispensable challenge of therapeutic resistance was combinatorial therapy, which involves the synergistic application of TK targeted therapeutics alongside other classes of inhibitors and naturally derived bioactive compounds [40, 150]. The strategy of combination therapy has delivered an outstanding therapeutic outcome in combatting serious cancers including breast [162–165]. Such novel combinations with TK targeted therapeutics have also displayed excellent DCR, increased PFS and better anti-tumor effect in preliminary phases of clinical trials [11, 109, 121]. Recently, contemporary pioneering investigations have employed RTK peptide vaccines as a therapeutic approach for TNBC immunization utilizing the AE37 peptide, a modified iteration of the naturally occurring AE36 wild-type peptide (HER2 776–790) derived from the intracellular domain of HER2 has demonstrated efficacy in patients with TNBC [166]. The observed DFS rate in TNBC patients subjected to vaccination was 77.7%, in contrast to the control TNBC population with a rate of 49.0% [166]. Brown et al. [136] documented that immunization with the AE37 peptide and GM-CSF in TNBC patients yielded a CBR rate of 85.7%, a notable contrast to the 36.4% observed in the control group. Significantly, this intervention showcased an earlier attainment of the DFS plateau, a status sustained over the 10-year span of follow-up [136]. These findings suggest that the AE37 peptide in combination with GM-CSF has the potential to augment the immunologic response against this particular subtype and may hold promise either independently or in combination with other therapeutic agents. Subsequent randomized investigations of AE37 in TNBC patients are warranted to further elucidate its clinical impact. It is also imperative to conduct phase III clinical trials involving these peptide vaccines in large TNBC cohorts to ascertain their potential benefits. Additionally, such studies should be extended to encompass various RTKs, particularly for patients displaying receptor mutations and diminished expression, in order to comprehensively address the diverse molecular profiles within this context.

## Role of RTKs/nRTKs in precision medicine paradigms: tailoring therapies for molecularly heterogeneous TNBC subtypes

TNBC constitutes a heterogeneous malady, primarily linked to pre-menopausal status and germline mutations in *BRCA1/2* [167]. Despite the widespread utilization of RTK and nRTK inhibitors for TNBC treatment, their efficacy has been thwarted in phase III clinical trials due to the intrinsic heterogeneity of the disease [47, 168]. Addressing these challenges necessitates innovative approaches. One potential solution lies in the realm of personalized and precision medicine, an approach advocating therapies tailored to the genetic and molecular signatures of individual patients [169–171]. While TNBC has traditionally been characterized as a distinct entity based on IHC features, specifically the absence of ER, PR, and HER2^-^ molecularly, it exhibits heterogeneity with diverse gene expression patterns [170]. Lehman et al. [45] identified distinct subtypes within TNBC, including BL1, BL2, IM, M, MSL, and LAR. Each subtype manifests unique gene expression patterns: BL1 and BL2 exhibit elevated expression of cell cycle and DNA damage response genes; IM subtype is enriched for genes associated with immune processes; M and MSL display enhanced expression of epithelial-mesenchymal transition genes and growth factor pathways; and LAR subtype is characterized by androgen receptor signaling. Consequently, the distinct molecular profiles of each TNBC subtype highlight the imperative need for tailored therapeutic approaches and endorse the application of personalized medicine. This section provides in-depth insight into the efficacy of RTK/nRTK inhibitors in personalized medicine, particularly when guided by the molecular signatures of TNBC patients.

Research elucidating targeted therapies for breast cancer has paved the way for precision medicine. Notably, PARP inhibitors have proven advantageous for individuals harboring *BRCA* germline mutations, showcasing advancements in recent years [172, 173]. However, their efficacy in treating metastatic TNBC remains suboptimal due to the limited prevalence of *BRCA* germline mutations within this subgroup [174, 175]. Novel ADCs, specifically sacituzumab govitecan and fam-trastuzumab deruxtecan-nxki (an HER2-directed antibody and topoisomerase inhibitor conjugate), have recently obtained FDA approval for patients with metastatic TNBC and metastatic HER2 positive breast cancer, respectively [167, 176, 177]. Additionally, gene fusions involving neurotrophin receptor tyrosine kinases, encompassing NTRK1, NTRK2, and NTRK3, lead to overexpression and constitutive activation of these genes and subsequent tumor growth [167, 178]. Although these events occur in approximately 1% of all solid tumors and less than 1% of all breast cancers, they represent a distinctive therapeutic target [167, 178]. The efficacy of larotrectinib, a tropomyosin receptor kinase (TRK) inhibitor, in TRK-fusion positive patients, including a breast cancer patient in a phase I/II trial, demonstrated promising outcomes [179]. Recent clinical trials, namely ALKA-372-001, STARTRK-1, and STARTRK-2, evaluated the anti-tumor efficacy and safety of entrectinib, another TRK inhibitor, in patients with solid tumors displaying TRK gene fusions. The results revealed favorable responses in patients with TRK fusions, including an overall response rate of 57%, a complete response of 7%, and a partial response of 50%, with a median duration of response of 10 months. Among these patients, 11% had breast cancer [167, 180]. Based on these findings, both larotrectinib and entrectinib have secured FDA and European Medicines Agency (EMA) approval for solid tumor patients with TRK gene fusions, showcasing high anti-tumor efficacy across various tumor subtypes and patient age groups [167]. While ongoing long-term clinical trials such as STARTRK-2 and NAVIGATE aim to provide more intricate insights into the use of TRK gene fusions in treating patients, it is crucial to emphasize the necessity for further validation of the TRK fusion status in TNBCs and metastatic TNBCs [167]. Additionally, the evidence supporting the efficacy of larotrectinib and entrectinib warrants continued scrutiny in TNBC subtypes.

The Fudan University Shanghai Cancer Center TNBC Umbrella (FUTURE) trials recently investigated the feasibility and clinical efficacy of subtyping-based precision therapy in refractory and heavily pre-treated metastatic TNBC patients [175, 181]. The FUTURE trial with clinical identifier NCT03805399 explored the utilization of molecular subtypes in treating refractory TNBC patients (*n* = 69) with a median of 3 previous lines of therapy [181]. In the next FUTURE study, the same team explored the utilization of molecular subtypes for treating heavily pre-treated metastatic TNBC patients [175]. In these studies, patients were stratified into distinct arms according to their TNBC subtypes and molecular features, resulting in various treatment approaches: A) LAR subtype with *HER2* mutations, pyrotinib with capecitabine, B) LAR subtype without *HER2* mutations, androgen receptor inhibitor with anti-CDK4/6 therapy, C) IM subtype, anti-PD-1 with Nab-paclitaxel, D) Basal-like immune-suppressed (BLIS) with *BRCA1/2* germline mutations, PARP inhibitor backbone therapy, E) BLIS without *BRCA1/2* mutations, anti-VEGF/VEGFR backbone therapy, F) Mesenchymal-like (MES) without *PI3K/Akt* mutations, VEGFR inhibitor backbone therapy, and G) MES with *PI3K/Akt* mutations, mTOR inhibitor with Nab-paclitaxel [175, 181]. These trials established a subtyping platform to guide precision medicine, categorizing TNBC patients based on their molecular landscape rather than single gene alterations [175, 181]. In the clinical trial conducted by Jiang et al. [181], ORR and DCR among the cohort of 69 subjects under investigation were determined to be 29.0% and 42.0%, respectively. Notably, arms C, IM subtype, anti-PD-1 with Nab-paclitaxel and E, BLIS without *BRCA1/2* mutations treated with anti-VEGF/VEGFR backbone therapy, which selectively addressed the immunotherapy targeting the IM and VEGFR therapy directed at the *BRCA1/2* gene wild type-BLIS subtypes, respectively, exhibited enhanced enrollment and demonstrated favorable therapeutic outcomes. In the study conducted by Liu et al. [175], with 141 heavily pre-treated TNBC patients enrolled, the study employed Bayesian predictive probability to enhance flexibility in sample size adequacy for each treatment cohorts, facilitating efficient evaluation of drug combination efficacy. Notably, the study demonstrated encouraging outcomes, with an ORR of nearly 30%, a median PFS of 3.4 months, and a median OS of 10.7 months-outperforming traditional chemotherapy outcomes in heavily pretreated TNBC patients [175]. Particularly, the investigation revealed the clinical benefits of RTK/nRTK inhibitors, in treating specific TNBC subtypes such as BLIS without *BRCA1/2* mutations and LAR subtype [175]. BLIS subtype is characterized by enhanced expression of the VEGF signature, indicative of tumor angiogenesis and a poor prognosis [175, 182]. Additionally, anti-VEGF/VEGFR backbone therapy, focusing on BLIS without *BRCA* germline mutations, resulted in a confirmed overall response rate approaching 30%, surpassing previously reported outcomes in heavily pretreated TNBC patients [69, 175]. These findings suggest preliminary efficacy of anti-VEGF/VEGFR therapy in BRCA wild-type BLIS tumors, warranting further exploration in *BRCA*-mutated patients [175]. Notably, combining bevacizumab or low-dose apatinib with VP-16 may be better tolerated than apatinib at a dose of 500 mg. Intriguingly, treatment groups A, i.e., LAR subtype with *HER2* mutations treated with pyrotinib with capecitabine and G arm, i.e., MES with *PI3K/Akt* mutations treated with mTOR inhibitor with Nab-paclitaxel, demonstrated promising outcomes in a small sample size. In the context of rare instances (2–4%), where metastatic breast cancer patients exhibit *ERBB2* mutations but are HER2-negative according to clinical guidelines, arm A, i.e., LAR subtype with *HER2* mutations treated with pyrotinib with capecitabine achieved a remarkable confirmed ORR of 75% [175]. This result implies the potential efficacy of anti-HER2 therapy in tumors harboring *HER2* mutations. Parallel findings from the SUMMIT study indicate that neratinib combined with trastuzumab exhibited significant anti-tumor activity in *ERBB2*-mutated TNBC patients after multiline therapy, achieving an ORR of 33.3% and a median PFS of 6.2 months [175, 183]. These observations indicated the clinical benefits of RTK/nRTK inhibitors, specifically tailored to particular TNBC subtypes. Of note, the clinical trial exploring the molecular pathway for metastatic TNBC in first-line treatment FUTURE-Trop2 (clinical identifier: NCT05928780) and the randomized control umbrella trial, FUTURE-SUPER (clinical identifier: NCT04395989) are currently in progress. Consequently, further exploration is warranted for other RTK/nRTK inhibitors based on the molecular subtypes of TNBC, emphasizing the need for personalized therapeutic approaches in the management of this complex disease. The FUTURE trial not only demonstrated the clinical feasibility of TNBC subtyping combined with next-generation sequencing but also highlighted the introduction of new biomarker-driven treatment groups based on patients’ molecular characteristics. The study’s dual-directed therapeutic strategy, guided by subtype and genomic characteristics, yielded promising efficacy and manageable toxicity. Moreover, integrated genomic and clinicopathological profiling provided insights into treatment efficacy associations, enabling the testing of novel ADCs for treatment cohorts with unsatisfactory responses.

As an ongoing platform for novel targeted regimens, pilot studies like the FUTURE trial showcase the potential for efficient testing of new drug–biomarker combinations within the context of TNBC subtyping. These endeavors generate valuable insights for further validation in expansion trials, emphasizing the significance of subtyping-based and genomic sequencing-guided strategies in achieving promising efficacy with manageable toxicity in heavily pre-treated metastatic TNBC patients.

## Conclusions

Distinguished by a spectrum of heterogeneous variants, breast cancer poses a substantial global health concern. This intricate nature is particularly pronounced in its subtype, TNBC, which encompasses 6 genetically distinguishable variants. The intricacies of these variants have contributed to the inadequacy of many existing TNBC therapeutics, prompting researchers to explore and elevate therapeutic modalities to a more advanced level with a specific emphasis on personalized care. In recent times, considerable endeavors have been undertaken to broaden the treatment prospects available for TNBC. TKs and their associated receptors have emerged as promising targets for therapeutic intervention, exhibiting promising outcomes in clinical trials. VEGF and its cognate receptor have been subjected to extensive investigation, with a discernible trend favoring the blockade of angiogenic factors in TNBC patients. Notably, bevacizumab and apatinib have demonstrated affirmative outcomes across multiple studies, substantiating their safety and efficacy profiles. The emergence of multi-targeting TKIs has unveiled an alternative avenue for augmenting survival probabilities. Numerous ongoing clinical trials are poised to unveil results that will further illuminate the trajectory of precision therapeutics against TNBC. However, additional clinical validation is required to ascertain the efficacy of the proposed TK targeted therapeutics, particularly with a larger TNBC patient population.

In our examination of published clinical trials, a notable observation emerges: the majority of phase III trials with TK targeted therapeutics involving larger patient cohorts have concluded without successfully enhancing the therapeutic outcomes for TNBC. The potential cause for this lack of success may lie in genomic variations. This is exemplified by a phase III trial involving bevacizumab, which initially demonstrated improved therapeutic outcomes in the TNBC cohort. Regrettably, upon long-term follow-up of the participating TNBC individuals, the results proved to be contrary. Additionally, the E2100 phase III trial revealed the inability of bevacizumab to augment paclitaxel treatment in a patient subgroup with VEGF-A amplification. Subsequent trials, involving larger study populations, further emphasizes the inefficacy of bevacizumab in treating TNBC patients. However, it is essential to note that the same mAb, when administered in conjunction with neoadjuvant treatment featuring taxanes, substantially increased the pCR exclusively in TNBC patients with *BRCA1/2* mutations. These findings highlight the significant impact of the genomic diversity of the TNBC patient population on the outcomes of clinical trials. Consequently, it becomes imperative to establish specific criteria for the selection of TNBC patients, based on comprehensive research and an understanding of the molecular interactions and interplay of TKs in TNBC. This necessitates a deeper exploration of preclinical evidence to enhance our understanding of TKs in the context of TNBC.

The implementation of more customized and precise methodologies, such as immunization employing RTK/nRTK peptides, as well as the utilization of ADCs, have demonstrated noteworthy therapeutic benefits for patients afflicted with TNBC. A recent comprehensive umbrella clinical trial, the FUTURE interim analysis, has exemplified the effectiveness of integrating the molecular landscape of TNBCs with Next Generation Sequencing in the treatment paradigm for heavily pre-treated metastatic TNBC patients. Notably, this investigation yielded the highest response rates within these patient cohorts, a feat previously unattained through conventional chemotherapeutic interventions. Further, specific TNBC subtypes, namely BLIS lacking *BRCA* mutations and LAR subtype, have exhibited potential benefits through the strategic application of RTK/nRTK inhibitors. The aforementioned observations delineate the clinical advantages associated with RTK and nRTK inhibitors, precisely customized for distinct subtypes within TNBC. Subsequent to these findings, a compelling rationale emerges for an extended investigation into alternative RTK/nRTK inhibitors aligned with the molecular sub-classifications of TNBC. This potentiates the imperative for tailored therapeutic strategies in addressing the intricacies of this pathological condition.

Nonetheless, the current landscape highlights a dearth of targeted therapies focusing on the signaling pathways implicated in oncogenicity, particularly within the ambit of TNBC, characterized as the most lethal variant of breast cancer. An imperative urgency indicates the development of novel targeted therapeutics characterized by enhanced efficacy, specificity, and diminished toxicity, with the aim of significantly enhancing the disease prognosis associated with this malignancy.

## Data Availability

Not applicable.

## Abbreviations

ABL: Abelson
Anti-EGFR-ILs-dox: Anti-EGFR immunoliposomes loaded with doxorubicin
ADCs: Antibody–drug conjugates
AXL: Anexelekto
BL1: Basal-like 1
BL2: Basal-like 2
BCR: Break point cluster
BLIS: Basal-like immune-suppressed
BRCA: Breast cancer gene
BTK: Bruton’s TK
CBR: Clinical benefit rate
cCR: Clinical complete response
CD: Clusters of differentiation
CR: Complete response
CSFR: Colony stimulating factor receptor
CERS6: Ceramide synthase 6
DC: Disease control
DCR: Disease control rate
DFS: Disease-free survival
EGFR: Epidermal growth factor receptor
ER: Estrogen receptor
ErbB: Erythroblastic leukemia viral oncogene homologue
FDA: Food and Drug Administration
FGFR: Fibroblast growth factor receptor
FLT3: FMS related receptor tyrosine kinase 3
FYN: FYN protooncogene
G-CSF: Granulocyte colony-stimulating factor
GM-CSF: Granulocyte–macrophage colony-stimulating factor
HER2: Human epidermal growth factor receptor 2
HLA: Human leukocyte antigen
IGFR: Insulin-like growth factor receptor
IHC: Immunohistochemistry
IM: Immunomodulatory
IRFI: Invasive recurrence-free interval
LAR: Luminal androgen receptor
mAbs: Monoclonal antibodies
MAPK: Mitogen-activated protein kinase
MERTK: Mer receptor tyrosine kinase 3
MES: Mesenchymal-like
MET: Mesenchymal-epithelial transition factor
M: Mesenchymal
MSL: Mesenchymal stem-like
mTOR: Mammalian target of rapamycin
Nab-paclitaxel: Nanoparticle albumin-bound paclitaxel
NCT: Neoadjuvant chemotherapy
NPS: Nelipepimut-S
nRTK: Non-receptor tyrosine kinase
NSCLC: Non-small cell lung cancer
NF-κB: Nuclear factor kappa B
ORR: Objective response rate
OS: Overall survival
PARP: Poly (ADP-ribose) polymerase
pCDK: Phospho-cyclin dependent kinase
pCR: Pathological complete response
PD-1: Programmed cell death protein 1
PDGFR: Platelet derived growth factor receptor
PFS: Progression-free survival
PI3K: Phosphoinositide 3-kinase
PKC: Protein kinase C
PLC-γ: Phospholipase C-gamma
PR: Progesterone receptor
PTEN: Phosphatase and tensin homolog
RET: Rearranged during transfection
RON: Récepteur d’origine nantais
RTK: Receptor tyrosine kinase
SRC: SRC protooncogene
STAT3: Signal transducer and activator of transcription 3
TIE-2: Tunica interna endothelial cell kinase 2
TK: Tyrosine kinase
TKI: Tyrosine kinase inhibitors
TNBC: Triple-negative breast cancer
TRK: Tropomyosin receptor kinase
TYRO3: Tyrosine protein kinase receptor 3
VEGF: Vascular endothelial growth factor
VEGFR: Vascular endothelial growth factor receptor
YES: Yamaguchi sarcoma oncogene
